# Recent advances in theranostics and oncology PET: emerging radionuclides and targets

**DOI:** 10.1007/s12149-025-02090-z

**Published:** 2025-07-27

**Authors:** Tadashi Watabe, Kenji Hirata, Mami Iima, Masahiro Yanagawa, Tsukasa Saida, Akihiko Sakata, Satoru Ide, Maya Honda, Ryo Kurokawa, Kentaro Nishioka, Mariko Kawamura, Rintaro Ito, Koji Takumi, Seitaro Oda, Shunsuke Sugawara, Keitaro Sofue, Daiju Ueda, Shinji Naganawa

**Affiliations:** 1 Department of Radiology, Graduate School of Medicine, The University of Osaka, 2-2 Yamadaoka, Suita, Osaka 565-0871 Japan; 2 Institute for Radiation Sciences, The University of Osaka, Suita, Japan; 3 Department of Diagnostic Imaging, Faculty of Medicine, Hokkaido University, Nishi 7, Kita 15, Kita-ku, Sapporo, Hokkaido, 060-8638 Japan; 4 Department of Fundamental Development for Advanced Low Invasive Diagnostic Imaging, Nagoya University Graduate School of Medicine, Tsurumai-Cho 65, Showa-ku, Nagoya, Aichi 466-8550 Japan; 5 Department of Radiology, University of Tsukuba, 1-1-1 Tennodai, Tsukuba, Ibaraki 305-8575 Japan; 6 Department of Diagnostic Imaging and Nuclear Medicine, Graduate School of Medicine, Kyoto University, 54 Shogoin Kawahara-Cho, Sakyo-ku, Kyoto, 606-8507 Japan; 7 Department of Radiology, University of Occupational and Environmental Health, 1-1 Iseigaoka, Yahatanishi-ku, Kitakyushu, Fukuoka 807-8555 Japan; 8 Department of Diagnostic Radiology, Kansai Electric Power Hospital, 2-1-7, Fukushima, Fukushima-ku, Osaka, 553-0003 Japan; 9 Department of Radiology, Graduate School of Medicine, The University of Tokyo, 7-3-1, Hongo, Bunkyo-ku, Tokyo, 113-8655 Japan; 10 Radiation Oncology Division, Global Center for Biomedical Science and Engineering, Faculty of Medicine, Hokkaido University, Nishi 7, Kita 15, Kita-ku, Sapporo, Hokkaido 060-8638 Japan; 11 Department of Radiology, Graduate School of Medicine, Nagoya University, Tsurumai-Cho 65, Showa-ku, Nagoya, Aichi 466-8550 Japan; 12 Department of Radiology, Graduate School of Medicine and Dental Sciences, Kagoshima University, 8-35-1 Sakuragaoka, Kagoshima, 890-8520 Japan; 13 Department of Diagnostic Radiology, Faculty of Life Sciences, Kumamoto University, 1-1-1 Honjo, Chuo-ku, Kumamoto, 860-8556 Japan; 14 Department of Diagnostic Radiology, National Cancer Center Hospital, 5-1-1, Tsukiji, Chuo-ku, Tokyo, 104-0045 Japan; 15 Department of Radiology, Graduate School of Medicine, Kobe University, 7-5-2, Kusunoki-Cho, Chuo-ku, Kobe, Hyogo 650-0017 Japan; 16 Department of Artificial Intelligence, Graduate School of Medicine, Osaka Metropolitan University, 1-4-3, Abeno-ku, Osaka, Japan

**Keywords:** Theranostics, Positron emission tomography, Radioligand therapy, SSTR, PSMA, Astatine, Terbium

## Abstract

Theranostics, a novel integrated approach that combines cancer diagnosis and therapy by switching the radionuclide, has attracted growing attention. Various oncology PET probes other than FDG have been developed for the highly sensitive and precise detection of many types of cancer with the advancements in PET scanners, supporting the innovative development in theranostics. In therapeutic applications, radioligand therapy targeting somatostatin receptors (SSTR) and prostate-specific membrane antigen (PSMA) has already demonstrated significant clinical benefits. Terbium-161 (^161^Tb) has emerged as a new beta and Auger electron emitter, showing greater therapeutic efficacy compared to ^177^Lu. Alpha emitters, such as astatine (^211^At), are currently being evaluated in investigator-initiated clinical trials, with preliminary efficacy data reported for [^211^At]NaAt in patients with radioiodine-refractory thyroid cancer. Novel pan-tumor targeting agents, such as TROP-2, Nectin-4, LAT1, GPC-1, and EphA2, are also under development, and clinical translation of radioligand therapy is anticipated. These innovations in theranostics are expected to further broaden the scope of precision medicine in oncology.

## Introduction

In recent years, theranostics—a novel approach that integrates cancer diagnosis and therapy by switching the radionuclide used to label compounds targeting specific molecules—has attracted growing attention. The term"theranostics"is a portmanteau of"therapeutics"and"diagnostics,"representing a cutting-edge technology that combines molecular imaging with targeted therapy [1].

In nuclear medicine-based theranostics (radio-theranostics), the process begins with molecular imaging to confirm the expression of specific targets. This is followed by targeted radionuclide therapy aimed at treating systemic cancer lesions [2]. Therapeutic agents labeled with alpha- or beta-emitting radionuclides selectively bind to tumor-specific targets, enabling the delivery of potent radiation directly to cancer cells while minimizing damage to surrounding healthy tissue. Among these, alpha radiation is particularly noteworthy due to its high linear energy transfer (LET) and short path length, which enable it to cause significant cytotoxicity with minimal off-target effects [3]. This makes targeted alpha therapy a promising, patient-friendly treatment strategy with substantial potential.

At the same time, cancer treatment has changed dramatically with the advent of immune checkpoint inhibitors, which are now used as standard therapy for various types of cancer [4]. Although they are often administered to patients with advanced-stage disease, they have recently been applied as neoadjuvant therapy in certain cancers as well [5]. However, sufficient therapeutic effects are not always achieved, and adverse effects can be problematic [6]. Therefore, PET imaging is expected to play an important role in predicting treatment response and monitoring side effects. Furthermore, various PET probes have been developed, and the performance of PET scanners has improved, enhancing both spatial and temporal resolution [7]. Image reconstruction techniques have also been optimized using artificial intelligence (AI), allowing for PET images with even less noise [8]. In this way, advances in oncology PET are supporting its application to radioligand therapy as part of theranostics.

This review summarizes recent trends in oncology PET and theranostics, and highlights emerging radionuclides and molecular targets that have attracted increasing attention in recent years.

## Recent trends in oncology PET

### FDG–PET and immunotherapy-related imaging

[^18^F]FDG PET remains the standard modality for cancer staging and the diagnosis of recurrence and metastasis. The emergence of immune checkpoint inhibitors, such as anti-PD-1 antibodies (e.g., nivolumab) and anti-CTLA-4 antibodies (e.g., ipilimumab), has expanded the landscape of cancer therapy [4]. Studies have shown that [^18^F]FDG uptake correlates with PD-1/PD-L1 expression, and PET/CT may serve as a non-invasive tool for evaluating PD-1/PD-L1 expression [9, 10]. Moreover, FDG–PET/CT has proven useful in identifying immune-related adverse events (irAEs) by visualizing abnormal uptake in the thyroid, lungs, gastrointestinal tract, muscles, and skin [11].

Recently, new immuno-PET probe, [^18^F]AlF–NOTA–PCP2 have been developed and can effectively measure PD-L1 expression in patients with head and neck cancers, outperforming the conventional FDG PET [12]. [^18^F]AlF–NOTA–PCP2 uptake strongly correlated with PD-L1 levels, highlighting its potential to improve patient stratification and guide personalized treatment strategies. This research was selected as the images of the year at the Society of Nuclear Medicine and Molecular Imaging (SNMMI) 2025 Annual Meeting.

### Intratumoral heterogeneity and radiomics

Numerous studies have highlighted the utility of analyzing intratumoral heterogeneity for disease differentiation, gene mutation prediction, and prognosis [13, 14]. Li et al. demonstrated that FDG–PET-based heterogeneity analysis using visual assessment could distinguish solitary pulmonary tuberculosis from non-small cell lung cancer [15]. Similarly, heterogeneity parameters have been shown to predict epidermal growth factor receptor (EGFR) mutation status and treatment response in lung adenocarcinoma patients undergoing EGFR tyrosine kinase inhibitor therapy [16, 17]. However, it remains difficult to evaluate heterogeneity using simple volume of interest measurements in daily clinical practice, and future challenges will involve identifying indices that are easy to measure and incorporating them into image interpretation viewers.

Radiomics research is evolving to incorporate AI. Wei et al. developed a fusion model combining radiomics and deep learning to differentiate pancreatic ductal adenocarcinoma from autoimmune pancreatitis [18]. Machine learning models utilizing FDG–PET/CT-derived features have demonstrated high accuracy in tumor characterization, staging, and prediction of treatment response and outcomes [19]. Furthermore, deep learning has shown promise in distinguishing benign from malignant lesions and in recurrence prediction [20, 21]. Nevertheless, visual assessment remains prevalent in daily clinical practice. To standardize interpretation, the Deauville criteria and volumetric measures like metabolic tumor volume have been adopted [22, 23]. It has been reported that combining qualitative and quantitative approaches enhances diagnostic reliability in somatostatin receptor scintigraphy by measuring SUV while taking into account the range of physiological uptake in normal organs [24].

In malignant lymphoma, not only conventional parameters like SUVmax but also novel metrics such as Dmax—the maximum interlesion distance—have emerged as independent predictors of progression-free and overall survival (PFS and OS) [25]. In addition, lymph node-to-primary tumor SUV ratios have been linked to prognosis [26]. While these new indicators show promise alongside SUVmax, their clinical utility and robustness will require further validation in future studies.

### Advancements in dynamic 4D and sequential PET imaging

The recent adoption of silicon photomultiplier (SiPM)-based PET systems has enabled higher sensitivity and improved spatial resolution. These technological advances have made dynamic four-dimensional (4D) PET imaging, which incorporates time as an additional dimension and evaluates the temporal changes in radiotracer distribution, a feasible method for evaluation in clinical practice in addition to conventional static spatial image assessment [27].

Dynamic 4D PET has demonstrated clinical utility across various scenarios. For example, the net influx constant in FDG–PET has been shown to distinguish lymph node metastases in lung cancer [28]. Parametric imaging using Patlak Ki, which refers to the influx rate constant estimated using the Patlak graphical analysis method, in [^68^Ga]DOTATATE PET for patients with neuroendocrine tumors can highlight lesions with greater contrast than conventional SUV images, potentially reducing false-positive findings [29]. In prostate cancer, PSMA uptake has been observed to increase over time, and dual-timepoint imaging (0–5 min and 55–60 min post-injection) is under investigation, suggesting the potential to improve diagnostic accuracy [30, 31].

In addition, with the advent of total-body PET, it has become possible to obtain whole-body PET images of sufficient quality even with low-dose injections and without extending the acquisition time [32]. Using this technique, the feasibility of performing two types of PET imaging, such as [^18^F]FDG and [^68^Ga]FAPI-04, with low-dose administration in a single day has been demonstrated [33]. However, total-body PET is very expensive and remains difficult to make widely available at this time. Therefore, it may be necessary to utilize AI to optimize imaging protocols to reduce radiation exposure for sequential PET imaging with existing PET scanners.

### Emerging PET probes beyond FDG

Amino acid tracers such as [^11^C]Methionine, [^18^F]Fluciclovine (FACBC), and [^18^F]Fluoroethyltyrosine (FET) are used for brain tumor imaging [34–36]. For example, [^11^C]Methionine PET/CT enables simultaneous assessment of brain and extracranial lesions in breast cancer patients with brain metastases. Tumor-to-normal ratio (TNR), which is defined as the ratio of the radiotracer uptake in the tumor to that in the corresponding normal tissue, has been shown to be more reliable than SUVmax in differentiating recurrence from radiation necrosis [37]. The hypoxic PET probe [^18^F]Fluoromisonidazole (FMISO) PET uptake patterns differ between IDH-mutant and IDH–wild-type gliomas, suggesting potential as a non-invasive mutation biomarker [38]. With regard to the application for radiotherapy, somatostatin receptor ligand PET using [^68^Ga]DOTATATE has been reported to be useful for defining the radiation field for the treatment of meningiomas [39].

**Fig. 1:**
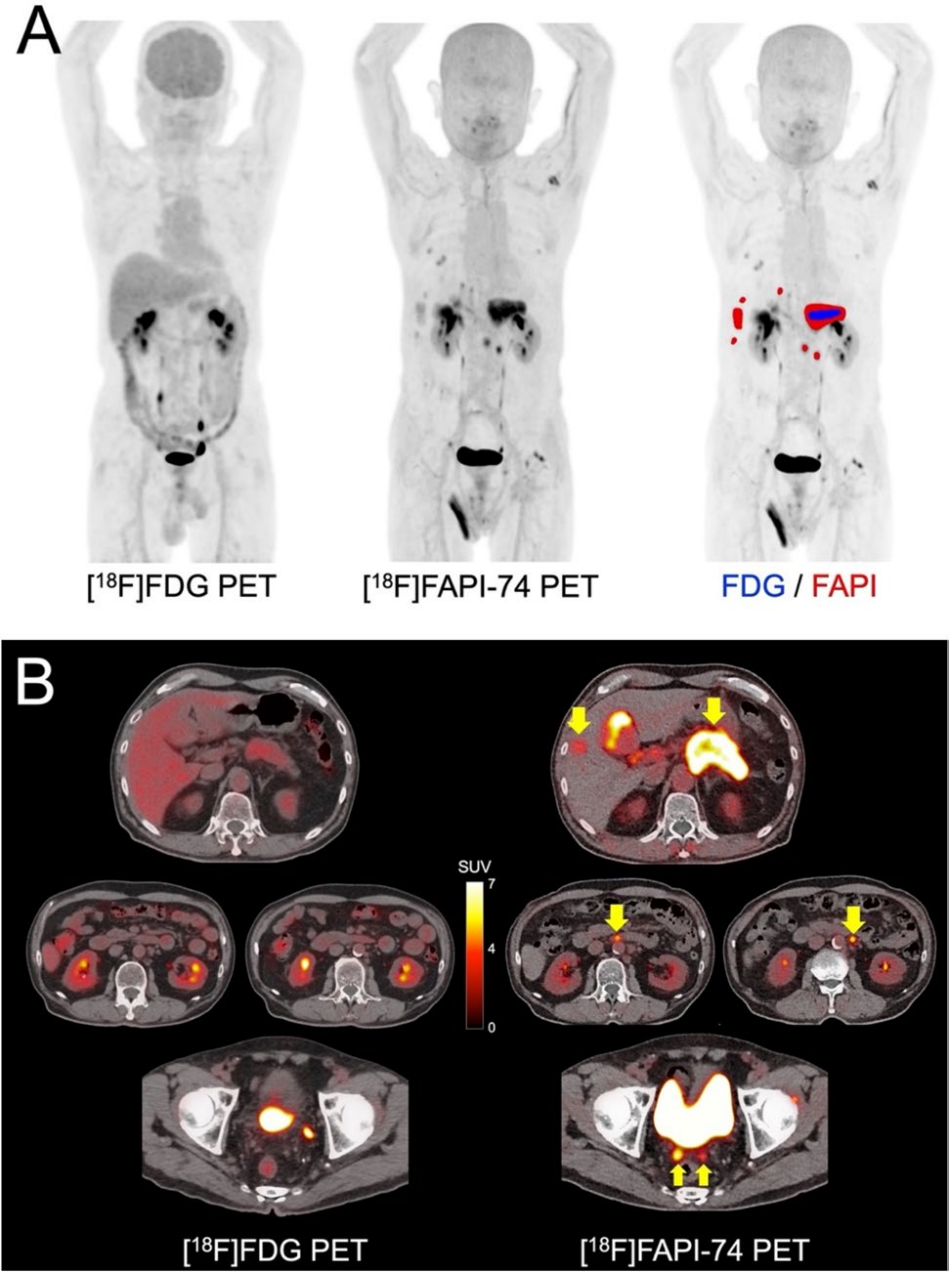
68-year-old male patient with multiple lymph node and peritoneal metastases of pancreatic cancer. **A** Comparison of the maximum intensity projection (MIP) images: the image on the right shows fusion with positive lesions on [^18^F]FAPI-74 PET (red-colored area) and [^18^F]FDG PET (blue-colored area), **B** PET/CT fusion images on [^18^F]FAPI-74 PET and [^18^F]FDG PET (arrows indicated metastatic lesions). [^18^F]FAPI-74 PET detected more metastatic lesions compared with [^18^F]FDG PET (SUVmax of the primary lesion is 9.4 and 3.2, respectively). (Cited from reference no. 40 in accordance with the open access policy.)

Fibroblast activation protein inhibitor (FAPI) PET, which targets cancer-associated fibroblasts, has demonstrated superior lesion detection and higher SUVmax in various cancers compared to FDG–PET [40] (Fig. 1). In a study that evaluated bone metastases of various cancers, SUVmax on [^68^Ga]FAPI–PET was an independent prognostic factor for OS [41]. FAPI–PET is also being investigated for liver fibrotic diseases beyond oncology application [42, 43].

**Fig. 2:**
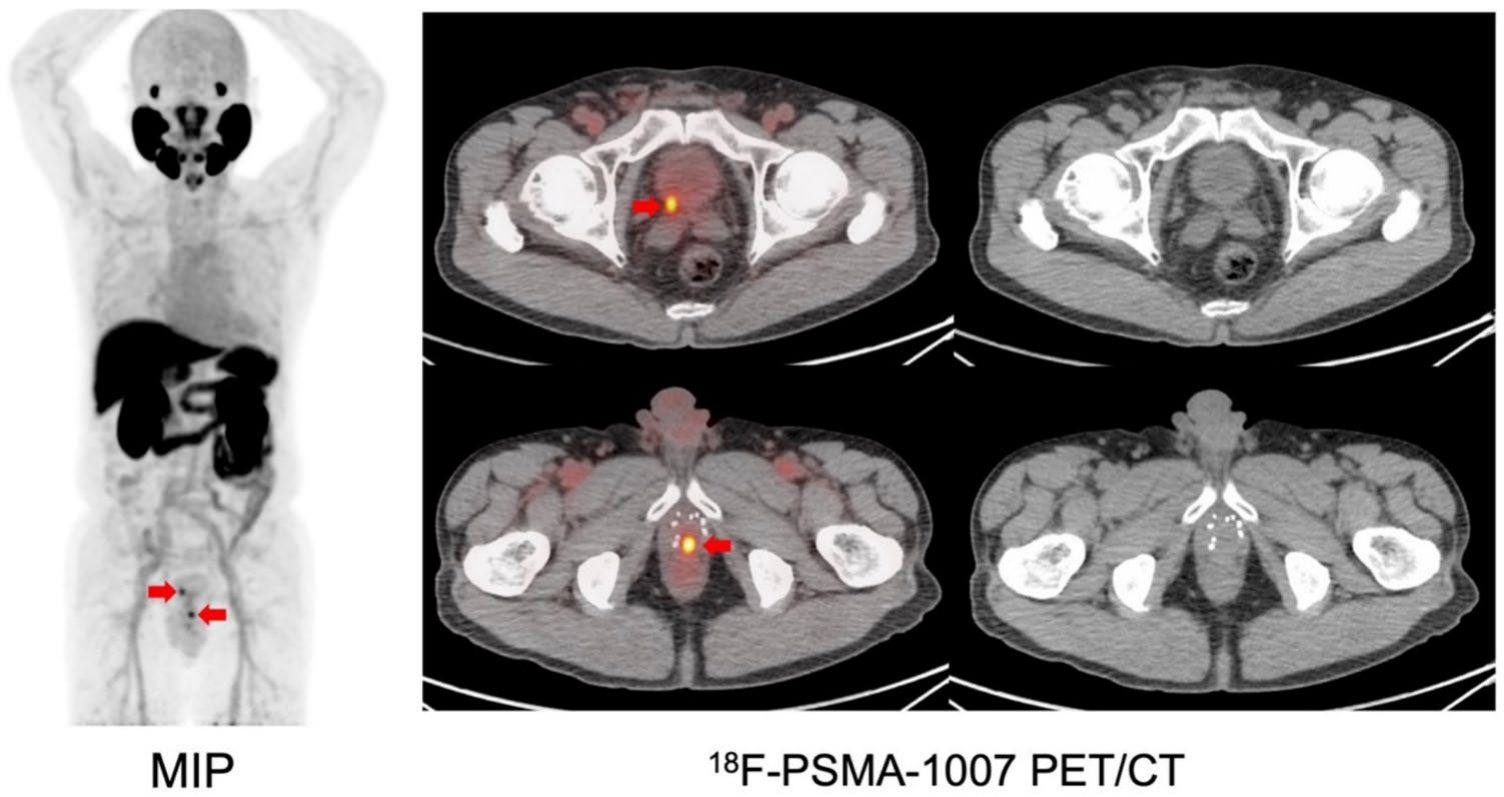
[^18^F]PSMA-1007 PET in patient with biochemical recurrence after ^125^I-seed implantation (PSA at PET: 3.32 ng/mL) (MIP: maximum intensity projection). Focal uptakes are observed on PSMA–PET (red arrows) and biopsy on the caudal lesion revealed a recurrence. Radiation therapy (cyber-knife) is performed targeting the two lesions and PSA value shows a decrease. (Cited from reference no. 46 in accordance with the open access policy.)

Prostate-specific membrane antigen (PSMA) PET is a highly sensitive imaging modality used across multiple stages of prostate cancer, including initial staging, detection of biochemical recurrence, and therapy planning [44, 45] (Fig. 2). Four PSMA tracers—[^68^Ga]PSMA-11, [^18^F]DCFPyL, [^18^F]PSMA-1007, and [^18^F]flotufolastat—have been approved in the U.S. or Europe. An international Phase III clinical trial (EAGLE-i trial), which assesses the diagnostic performance of [^18^F]PSMA-1007 PET in newly diagnosed, high-risk or very-high-risk prostate cancer patients in comparison with conventional imaging, is currently underway in Japan, following prior clinical research and Phase I/IIa studies [46, 47]. In addition, new ^99m^Tc-labeled SPECT probes are being developed to improve accessibility in regions where the availability of PET imaging is limited [48, 49]. Given the heterogeneity or absence of PSMA expression in some patients with castration-resistant prostate cancer (CRPC) [50], gastrin-releasing peptide receptor (GRPr) PET is also being investigated as a complementary imaging strategy [51].

In breast cancer, 16α-[^18^F]fluoro-17β-estradiol (FES)–PET has demonstrated higher radiotracer uptake and greater lesion detection rates compared to FDG–PET in estrogen receptor (ER)–positive breast cancer [52]. Furthermore, FES PET not only allows for whole-body assessment of ER expression but also serves as a valuable predictive biomarker for assessing the likelihood of response to endocrine therapy response [53]. FES–PET can help guide treatment decisions, monitor therapeutic efficacy, and potentially detect resistance early, thereby contributing to more personalized management strategies for patients with ER–positive breast cancer.

As described above, many promising PET probes targeting various molecules other than FDG have been developed for the highly sensitive and precise detection of cancer, and these probes are expected to be useful not only for diagnostic imaging but also for theranostics. Furthermore, FDG PET also plays a complementary role depending on tumor differentiation status and will not be completely replaced by emerging PET probes. Combined evaluation using dual [^68^Ga]DOTATATE and FDG PET/CT has been reported to be useful as a prognostic biomarker in metastatic gastroenteropancreatic neuroendocrine neoplasms [54].

## Recent trends in targeted radioligand therapy

### [^177^Lu]DOTATATE (Lutathera)

[^177^Lu]DOTATATE is a targeted radiopharmaceutical used for the treatment of somatostatin receptor (SSTR)–positive gastroenteropancreatic neuroendocrine tumors (GEP–NETs). The NETTER-1 trial demonstrated significantly prolonged PFS in midgut NETs, leading to FDA and PMDA approval [55]. The NETTER-2 trial showed that combining [^177^Lu]DOTATATE with octreotide long-acting release (LAR) significantly improved PFS in previously untreated advanced GEP–NETs [56]. In April 2024, FDA approval was extended to include pediatric patients aged 12 years and older.

In terms of predicting treatment efficacy, a retrospective analysis of 40 patients with NETs treated with [^177^Lu]DOTATATE demonstrated that visual and quantitative analyses of baseline SSTR–PET may be useful indicators for predicting treatment outcomes. A low mean whole-body SUV and presence of SSTR–PET-negative lesions on baseline SSTR–PET may predict shortened PFS [57].

### PSMA-targeted radioligand therapy

PSMA expression was observed on PSMA–PET in 95% of patients with metastatic castration-resistant prostate cancer (mCRPC) [58], indicating a promising target for radionuclide therapy. In the TheraP trial, [^177^Lu]PSMA-617 showed higher PSA response rates and fewer grade 3 or 4 adverse events compared to chemotherapy with cabazitaxel (66% vs. 44% PSA responses; grade 3–4 adverse events: 33% vs. 53%) [59]. In the VISION trial, [^177^Lu]PSMA-617 (Pluvicto) was shown to improve OS in patients receiving best supportive care after taxane chemotherapy for mCRPC (15.3 months vs. 11.3 months, HR 0.62), and was approved by the FDA in 2022 [60]. Furthermore, in the PSMAfore study, in patients with mCRPC who had not received taxane chemotherapy, [^177^Lu]PSMA-617 prolonged radiographic PFS compared to a change in androgen receptor pathway inhibitor (ARPI), with a favorable safety profile (11.6 months vs. 5.6 months, HR 0.49). FDA approval for its use prior to chemotherapy was granted in April 2025 [61]. Recently, [^177^Lu]PSMA-617 in combination with hormone therapy provided a statistically significant and clinically meaningful benefit in radiographic PFS in patients with PSMA–positive metastatic hormone-sensitive prostate cancer (mHSPC), compared to hormone therapy alone, based on interim results from the Phase 3 PSMAddition trial (NCT04720157). Thus, the indication for PSMA-targeted radioligand therapy (RLT) is being shifted earlier in the treatment sequence, expanding the number of eligible patients.

The validity of criteria for companion diagnostics using PSMA–PET in real-world clinical practice for PSMA-targeted RLT is also under evaluation. While the VISION trial used PSMA–PET/CT-based inclusion criteria, the TheraP trial employed dual-tracer imaging, including FDG–PET/CT. Retrospective application of the VISION trial’s inclusion criteria shows benefits in OS and PFS following PSMA–RLT, but the TheraP criteria may be too stringent for patients with advanced prostate cancer. Therefore, more relaxed eligibility criteria, such as those used in the VISION trial, may facilitate broader access to PSMA-targeted therapy [62].

### [^131^I]MIBG and radioiodine therapy

Metaiodobenzylguanidine (MIBG) is a norepinephrine analog that is used as both a diagnostic and therapeutic radiopharmaceutical, particularly in the management of pheochromocytoma, paraganglioma, and neuroblastoma [63]. Structurally similar to norepinephrine, MIBG is selectively taken up by adrenergic nerve terminals and tumor cells through the norepinephrine transporter. [^131^I]MIBG has been approved in Japan since 2022 for the treatment of pheochromocytoma and paraganglioma, with expanded coverage for MIBG–positive neuroblastoma beginning in April 2025. In pediatric patients with high-risk neuroblastoma, myeloablative therapy with [^131^I]MIBG in combination with high-dose chemotherapy and bone marrow transplantation has shown high efficacy, with 67% achieving complete remission [64].

Radioiodine (^131^I) is a standard treatment for differentiated thyroid cancer (DTC), used for the ablation of residual thyroid tissue after total thyroidectomy and for the treatment of distant metastatic disease, especially in patients with iodine-avid lesions [65]. Recently, a retrospective cohort analysis of patients from the SEER database showed that ^131^I therapy was associated with a survival benefit in DTC patients with distant metastasis [66]. In addition, adjuvant radioiodine therapy has been shown to be beneficial for achieving biochemical remission and prolonging disease-free survival in patients undergoing reoperation for cervical lymph node recurrence in papillary thyroid cancer. Patients who received ^131^I demonstrated significantly better biochemical remission rates than those who did not. It has been shown that adjuvant radioiodine therapy after reoperation for patients with recurrent or residual thyroid cancer may contribute to biochemical remission and extend disease-free survival to some extent [67].

A retrospective study examining the relationship between changes in serum thyroglobulin levels before and after radioiodine therapy and recurrence-free survival in patients with DTC showed that these changes were associated with radiographic PFS. Patients with decreased post-therapy thyroglobulin levels had a favorable prognosis, even if thyroglobulin remained detectable after ^131^I therapy [68].

Thus, while ^131^I therapy has been performed for many years, high-level evidence regarding its prognostic efficacy had been limited, but such evidence is now gradually accumulating. Although the optimal timing of postoperative ^131^I in DTC patients remains unclear due to a lack of definitive evidence [69], deferring initial radioiodine therapy beyond 180 days following total thyroidectomy may be associated with inferior survival outcomes in patients with differentiated thyroid cancer [70].

### Dosimetry and practical considerations

Dosimetry and post-therapeutic imaging are also important for monitoring and predicting treatment efficacy and side effects in RLT. In the future, it may be possible to achieve personalized medicine by determining the injection dose that maximizes therapeutic efficacy while minimizing major side effects through dosimetric calculation. Accurate dosimetry typically requires time-lapse imaging at multiple timepoints, but there is a recent trend toward simplified assessment using single-timepoint imaging [71]. However, since planar imaging alone tends to overestimate the absorbed dose, it has been reported that combining at least one SPECT acquisition is preferable [72].

In addition, in countries with strict radiation safety regulations, such as Japan and Germany, therapy using ^131^I or ^177^Lu requires hospitalization in an isolated radionuclide therapy room or a specially equipped treatment room, and securing such rooms is often a logistical challenge. To be discharged, patients must meet specific release criteria on radiation dose, and a practical prediction formula has been developed to estimate the dose rate reduction around patients after administration with [^177^Lu]DOTATATE. It has been reported that a predictive formula incorporating maximum tumor diameter and creatinine clearance shows a strong correlation with dose rate reduction and can serve as a useful tool for post-treatment patient management [73].

## Future outlook for theranostics

### Next-generation beta-emitter

﻿Currently, lutetium-177 (^177^Lu; half-life: 6.7 days) is the primary beta-emitting radioisotope used in the development of RLT agents, but terbium-161 (^161^Tb; half-life: 6.9 days), which has a similar half-life and beta radiation energy, is gaining increasing attention [74]. ^161^Tb emits not only beta particles but also Auger electrons and internal conversion electrons. Auger electrons deposit their energy within a few nanometers, causing dense ionization near DNA and the effective induction of DNA double-strand breaks when the radionuclide is internalized close to the cell nucleus [75]. As a result, [^161^Tb]PSMA-617 has shown greater therapeutic efficacy than [^177^Lu]PSMA-617 in both in vitro studies and tumor-bearing animal models [76].

In addition, in a preclinical study comparing an SSTR-targeting agonist (with internalization) and an antagonist (LM3; without internalization), labeling with ^161^Tb demonstrated superior therapeutic effects compared to ^177^Lu, suggesting that Auger electrons emitted by ^161^Tb may exert cytotoxic effects on the cell membrane [77]. Clinical use of ^161^Tb-labeled agents targeting PSMA and SSTR2 antagonists has already been reported [78, 79], with several clinical trials currently in progress. Their future outcomes are eagerly awaited.

### Targeted alpha therapy

**Fig. 3:**
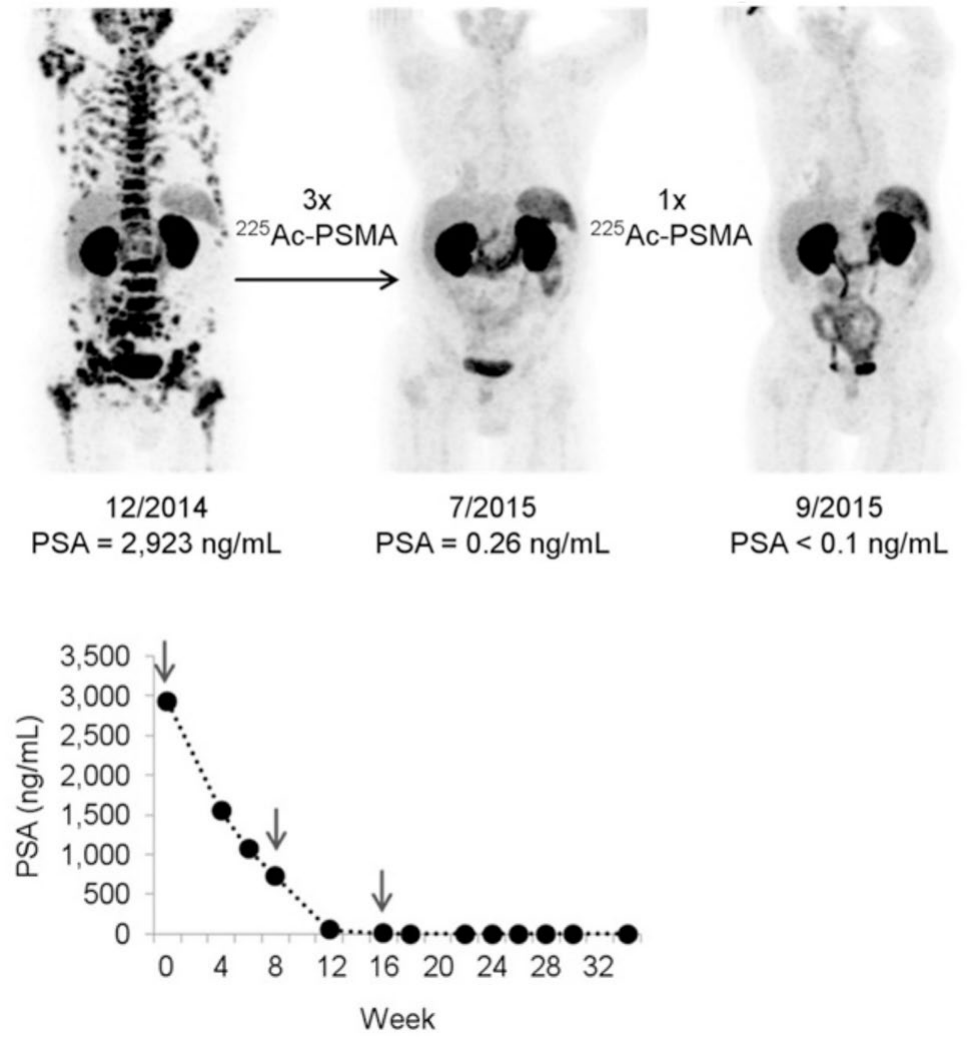
[^68^Ga]PSMA-11 PET/CT scans in a metastatic castration-resistant prostate cancer patient with diffuse bone metastases after administration of [^225^Ac]PSMA-617. Complete remission was achieved after four cycles of treatment. (Cited from reference no. 79 in accordance with the open access policy.)

Since alpha particles emit high energy over a short range, they are expected to be very effective in treating refractory patients who are resistant to conventional beta-ray therapy (Fig. 3) [80]. Among alpha-emitting radionuclides, radium-223 (Ra) is used for treating bone metastases in mCRPC, accumulating in osteoblastic lesions with increased bone turnover due to its calcium-mimetic properties. Although ^223^Ra was shown to extend OS in the ALSYMPCA trial [81], its therapeutic effect—such as PSA reduction—was limited, because it does not directly target tumor cells. However, the PEACE-3 study demonstrated that combination therapy with enzalutamide and ^223^Ra significantly prolonged OS (42.3 months vs. 35.0 months, HR 0.69) [82]. Fracture risk had been a concern with this combination, but it was confirmed that the use of bone-protecting agents did not increase the incidence of fractures [83]. Nevertheless, since ^223^Ra cannot be easily used as a radiolabeled compound, further development has been limited.

Currently, clinical trials are underway for targeted alpha therapy using three alpha-emitting radionuclides: actinium-225 (^225^Ac), lead-212 (^212^Pb), and astatine-211 (^211^At) (Table 1) [84]. Targeted alpha therapy with [^225^Ac]DOTATATE has shown promising results, improving overall survival even in patients who are refractory to prior [^1^⁷⁷Lu]DOTATATE therapy, with minimal toxicities in GEP–NETs [85]. In mCRPC patients, [^225^Ac]PSMA RLT has demonstrated that approximately 60% of patients achieve a PSA decline of more than 50%, and 70–80% of patients experience some PSA decline after therapy [86, 87].

**Table 1:** Comparison of recent therapeutic radionuclides with alpha or beta emission

	^177^Lu	^225^Ac	^212^Pb	^211^At
Radiation	β	α	α/(β)	α
Half-life	7 days	10 days	10.6 h	7.2 h
Therapeutic effect	+	+ +	+ +	+ +
Exposure to surroundings	High	Low	Low–moderate	Low
Isolation	Required	Not required	Not required?	Not required
Outpatient treatment	× ^*^	〇	〇?	〇
Domestic production	×	×	×	〇
Cyclotron manufacturing	×	Possible	×	〇
Imaging	〇	×	〇	〇
Approval status	FDA approved	No	No	No

+ : moderate therapeutic effect; + + : strong therapeutic effect,?: data or consensus currently limited or under investigation,〇: generally feasible; × : not generally feasible, *: depending on regulatory authority regulations in each country)

**Fig. 4:**
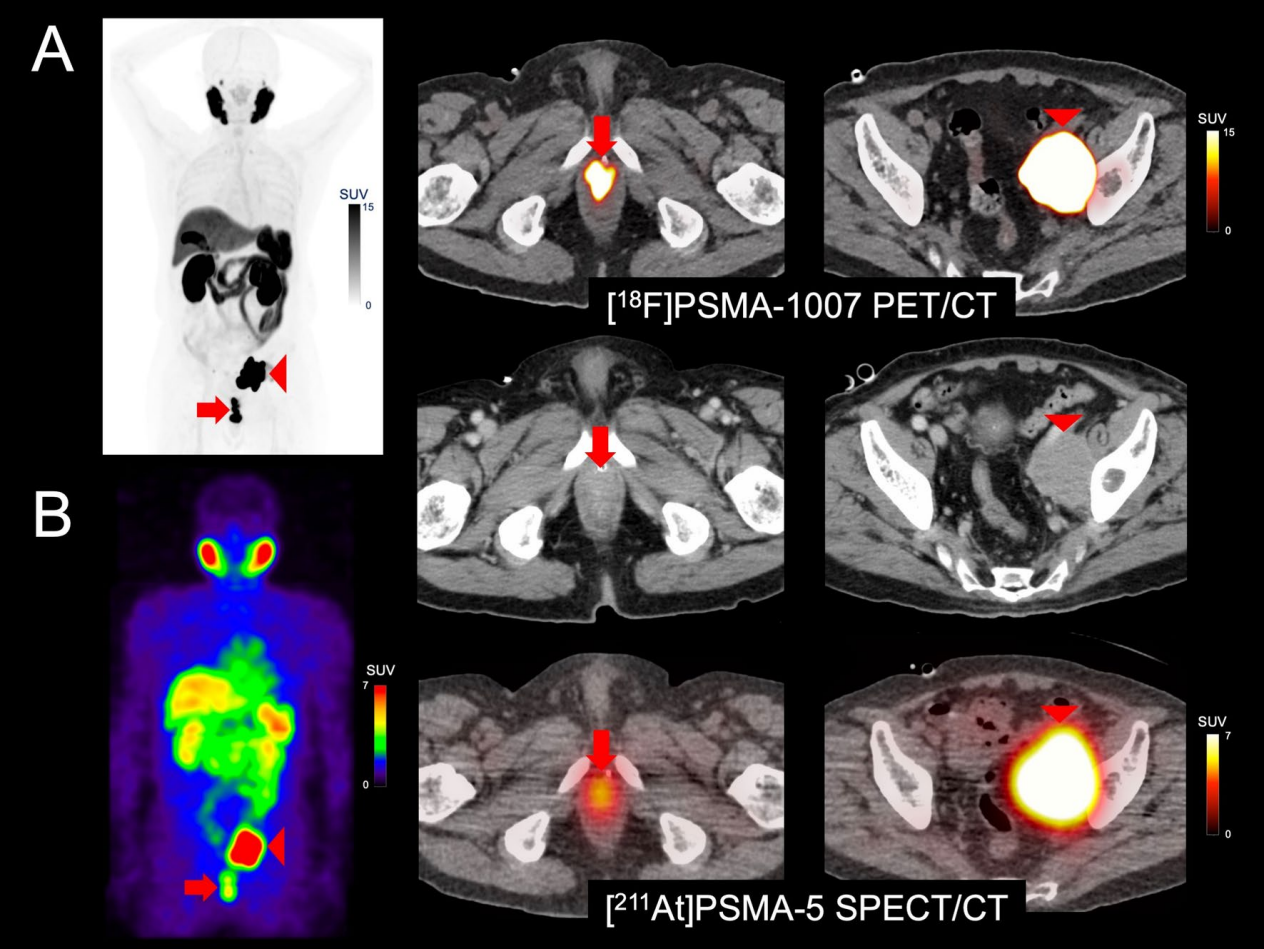
[^211^At]PSMA-5 was administered to a man in his 70 s with metastatic castration-resistant prostate cancer refractory to standard treatment, including androgen receptor signaling inhibitors, docetaxel, and cabazitaxel. Pre-treatment [^18^F]PSMA-1007 PET/CT (**A**) and [^211^At]PSMA-5 SPECT/CT (**B**) images showed similar distribution patterns, with high uptake in recurrent/metastatic lesions (left: maximum intensity projection, right: fusion and contrast-enhanced CT images). Both images revealed high accumulation in the soft tissue mass within the prostate area (SUVmax = 60.7 on [^18^F]PSMA-1007 PET and 4.9 on [^211^At]PSMA-5 SPECT) (arrows) and in the enlarged left external iliac lymph node metastasis (SUVmax = 143.7 and 17.6, respectively) (arrow heads). (Cited from reference no. 85 in accordance with the open access policy.)

In the case of astatine, Japan has taken a leading role, having completed an investigator-initiated clinical trial using sodium astatide ([^211^At]NaAt) (Alpha-T1 study: NCT05275946), and currently conducting trials with [^211^At]PSMA-5 (Alpha-PS1 study: NCT06441994) and [^211^At]MABG (jRCT2021220012) (Fig. 4) [88, 89]. In the clinical trial using [^211^At]NaAt, tolerability and evidence of efficacy were confirmed in patients with radioiodine-refractory thyroid cancer [90]. While the supply of alpha-emitting radionuclides has historically been a limiting factor, industrial production is now underway, and a manufacturing and distribution infrastructure is being established, raising expectations for large-scale availability in the near future [84].

### Emerging molecular targets

Development is also underway for new targets in RLT. Carbonic anhydrase IX (CA IX) is a cancer-associated enzyme activated by hypoxia on cell membranes in various tumors, supporting pH regulation as well as cancer cell invasion and metastasis [91]. It catalyzes the reversible conversion of carbon dioxide to bicarbonate and protons, and interacts with various molecules that transport ions and metabolites across cell membranes [91]. CA IX is highly expressed in clear cell renal cell carcinoma, and earlier development efforts primarily focused on antibodies, such as [^89^Zr]-DFO–girentuximab [92]. The recent emergence of small molecules and peptides has enabled the initiation of clinical research and trials using [^68^Ga]DPI-4452 PET, [^64^Cu]PD-32766 PET, and [^177^Lu]DPI-4452 (NCT05706129) [93]. In [^68^Ga]-DPI-4452 PET, some metastatic lesions showed SUVmax exceeding 100, suggesting promising uptake characteristics; however, the correlation with the therapeutic efficacy of [^177^Lu]DPI-4452 remains to be conclusively demonstrated.

**Fig. 5:**
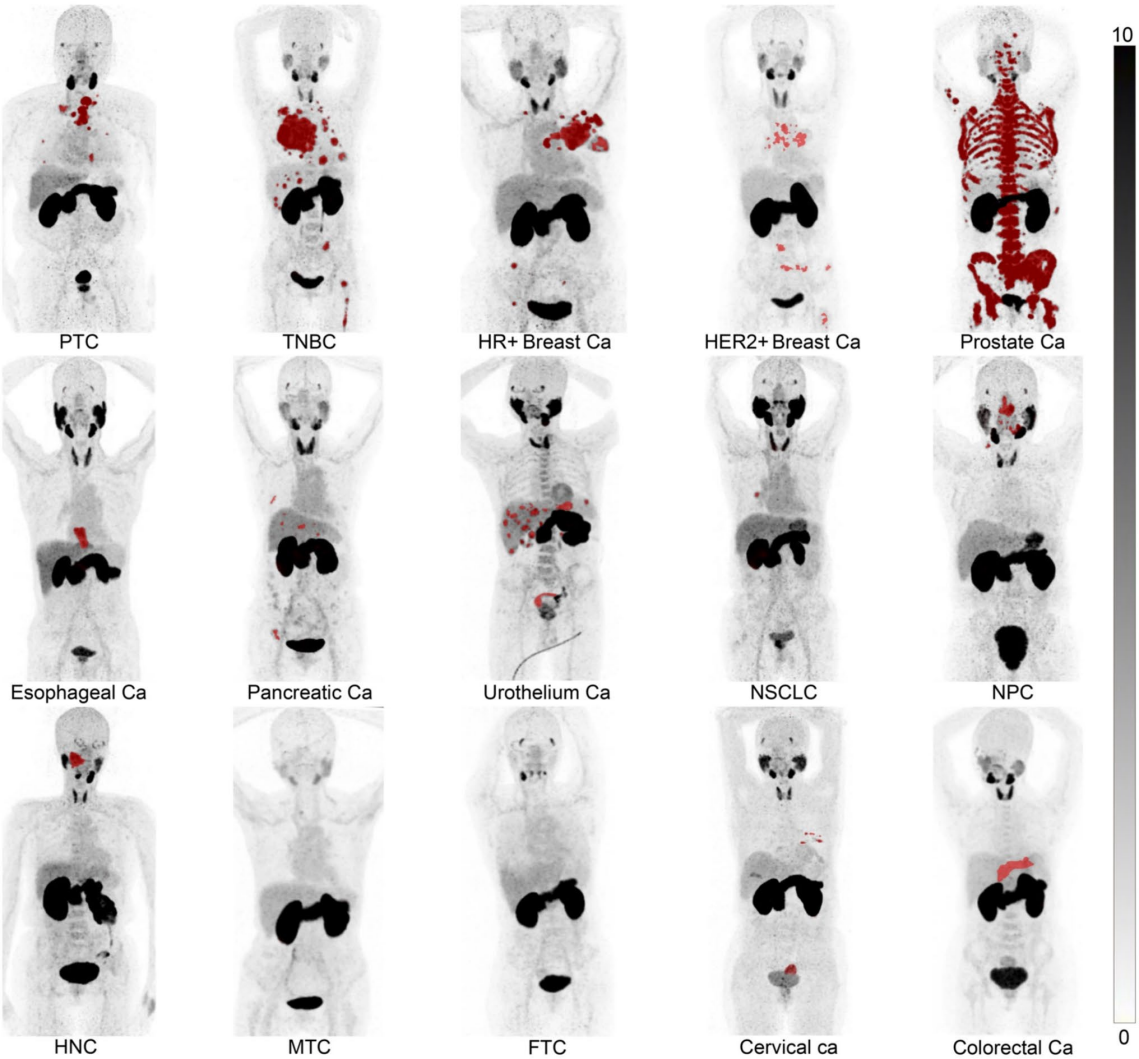
[^68^Ga]MY6349 PET/CT imaging targeting TROP-2 in patients with 15 different histologically confirmed tumor entities. Ca, cancer; FTC, follicular thyroid carcinoma; HER2, human epidermal growth factor receptor 2; HNC, head and neck cancer; HR, hormone receptor; MTC, medullary thyroid cancer; NPC, nasopharyngeal carcinoma; NSCLC, non-small cell lung cancer; PTC, papillary thyroid carcinoma; TNBC, triple-negative breast cancer. (Cited from reference no. 92 in accordance with the open access policy.)

Trophoblast cell surface antigen 2 (TROP-2) is a transmembrane glycoprotein highly expressed in various solid tumors and is involved in cell adhesion and signal transduction [94]. It promotes tumor formation and metastasis and is overexpressed in many cancers, including pancreatic, breast, colorectal, bladder, and non-small cell lung cancers, while showing minimal expression in normal tissues [95]. An anti-TROP-2 antibody–drug conjugate (ADC) has already been approved for triple-negative breast cancer. In addition, [^68^Ga]MY6349 PET, developed by Chen et al., has demonstrated high uptake in various cancer types, including breast cancer (Fig. 5) [96]. It has also been shown that [^68^Ga]MY6349 PET/CT can detect early therapeutic response to ADC treatment in triple-negative breast cancer [96]. ^89^Zr/^177^Lu-labeled TROP-2 antibodies have also been developed and evaluated in preclinical studies [97]. Clinical translation using small molecules or peptides for TROP-2-targeted RLT is anticipated in the near future.

Nectin-4 is a transmembrane protein and a member of the cell adhesion molecule family that interacts with other Nectin family members (especially Nectin-1), thereby enhancing intercellular adhesion [98]. In the tumor microenvironment, it promotes cell proliferation, migration, and invasion, and is highly expressed in various epithelial tumors, including bladder, lung, breast, and head and neck cancers. Conversely, in normal tissues, it is expressed only at low levels in select skin, lung, and placental tissues. A Nectin-4-targeted ADC (enfortumab vedotin) has already been approved for unresectable urothelial carcinoma that has progressed after chemotherapy [99]. [^68^Ga]N188 PET targeting Nectin-4 has shown high accumulation in lesions, correlating with Nectin-4 expression levels as confirmed by immunohistochemistry in patients with advanced urothelial carcinoma [100]. [^68^Ga]N188 PET imaging has also been reported in a small cohort of patients with various types of cancer, suggesting its potential for theranostics application [101].

Furthermore, PET imaging and therapeutic agents targeting pan-tumor molecules such as LAT1, GPC-1, and EphA2 have already been developed and are currently undergoing optimization for clinical translation [102–104]. PET probes that demonstrate high tumor accumulation and detection sensitivity—such as those used in PSMA theranostics—also hold great promise as therapeutic agents. These highly promising theranostic radioligands are expected to be incorporated into future clinical practice.

## Summary

This review highlights recent advancements in oncology PET imaging and theranostics, with a focus on emerging radionuclides and molecular targets. As the field of theranostics continues to evolve, these innovations are expected to further broaden the scope of precision cancer diagnosis and treatment.

## References

[1] Weber WA, Barthel H, Bengel F, Eiber M, Herrmann K, Schäfers M. What Is Theranostics? J Nucl Med. 2023;64:669–70. 37055220 10.2967/jnumed.123.265670

[2] Al-Ibraheem A, Scott AM, Abdlkadir AS, Vrachimis A, Lamoureux F, Trujillo PB, et al. Consensus nomenclature for radionuclide therapy: initial recommendations from nuclear medicine global initiative. J Nucl Med. 2025;66:757–63. 40147850 10.2967/jnumed.124.269215

[3] Feuerecker B, Kratochwil C, Ahmadzadehfar H, Morgenstern A, Eiber M, Herrmann K, et al. Clinical translation of targeted α-therapy: an evolution or a revolution? J Nucl Med. 2023;64:685–92. 37055224 10.2967/jnumed.122.265353

[4] Wei J, Li W, Zhang P, Guo F, Liu M. Current trends in sensitizing immune checkpoint inhibitors for cancer treatment. Mol Cancer. 2024;23:279. 39725966 10.1186/s12943-024-02179-5 PMC11670468

[5] Villacampa G, Navarro V, Matikas A, Ribeiro JM, Schettini F, Tolosa P, et al. Neoadjuvant immune checkpoint inhibitors plus chemotherapy in early breast cancer: a systematic review and meta-analysis. JAMA Oncol. 2024;10:1331–41. 39207778 10.1001/jamaoncol.2024.3456 PMC12422158

[6] Jayathilaka B, Mian F, Franchini F, Au-Yeung G, IJzerman M,. Cancer and treatment specific incidence rates of immune-related adverse events induced by immune checkpoint inhibitors: a systematic review. Br J Cancer. 2025;132:51–7. 39489880 10.1038/s41416-024-02887-1 PMC11723908

[7] Hirata K, Kamagata K, Ueda D, Yanagawa M, Kawamura M, Nakaura T, et al. From FDG and beyond: the evolving potential of nuclear medicine. Ann Nucl Med. 2023;37:583–95. 37749301 10.1007/s12149-023-01865-6

[8] Hirata K, Matsui Y, Yamada A, Fujioka T, Yanagawa M, Nakaura T, et al. Generative AI and large language models in nuclear medicine: current status and future prospects. Ann Nucl Med. 2024;38:853–64. 39320419 10.1007/s12149-024-01981-x PMC11813999

[9] Shen LF, Fu ZM, Zhou SH. The role of radiotherapy in tumor immunity and the potential of PET/CT in detecting the expression of PD-1/PD-L1. Jpn J Radiol. 2024;42:347–53. 37953364 10.1007/s11604-023-01507-x

[10] Hughes DJ, Josephides E, O’Shea R, Manickavasagar T, Horst C, Hunter S, et al. Predicting programmed death-ligand 1 (PD-L1) expression with fluorine-18 fluorodeoxyglucose ([^18^F]FDG) positron emission tomography/computed tomography (PET/CT) metabolic parameters in resectable non-small cell lung cancer. Eur Radiol. 2024;34:5889–902. 38388716 10.1007/s00330-024-10651-5 PMC11364571

[11] Gideonse BM, Birkeland M, Vilstrup MH, Grupe P, Naghavi-Behzad M, Ruhlmann CH, et al. Organ-specific accuracy of [^18^F]FDG-PET/CT in identifying immune-related adverse events in patients with high-risk melanoma treated with adjuvant immune checkpoint inhibitor. Jpn J Radiol. 2024;42:753–64. 38504000 10.1007/s11604-024-01554-y PMC11217074

[12] Wang Y, Liu Z, Hu M, Yu J. Can [18F]FDG-PET/CT predict PD-L1 expression in head and neck carcinoma? a head-to-head comparison with a novel pd-l1 pet tracer. J Nucl Med. 2025;66(supplement 1): 251095.

[13] Gadens Zamboni C, Dundar A, Jain S, Kruzer M, Loeffler BT, Graves SA, et al. Inter- and intra-tumoral heterogeneity on [^68^Ga]Ga-DOTA-TATE/[^68^Ga]Ga-DOTA-TOC PET/CT predicts response to [^177^Lu]Lu-DOTA-TATE PRRT in neuroendocrine tumor patients. EJNMMI Rep. 2024;8:39. 39613925 10.1186/s41824-024-00227-3 PMC11607192

[14] Smeets EMM, Trajkovic-Arsic M, Geijs D, Karakaya S, van Zanten M, Brosens LAA, et al. Histology-based radiomics for [^18^f]fdg pet identifies tissue heterogeneity in pancreatic cancer. J Nucl Med. 2024;65:1151–9. 38782455 10.2967/jnumed.123.266262

[15] Li Q, Li Y, Yuan H, Yang F, Huang Y, Song X, Jiang L. PET morphology helps distinguish solitary and solid pulmonary tuberculosis from non-small cell lung cancer. Jpn J Radiol. 2023;41:312–21. 36227458 10.1007/s11604-022-01351-5

[16] Ni M, Wang S, Liu X, Shi Q, Zhu X, Zhang Y, et al. Predictive value of intratumor metabolic and heterogeneity parameters on [^18^F]FDG PET/CT for EGFR mutations in patients with lung adenocarcinoma. Jpn J Radiol. 2023;41:209–18. 36219311 10.1007/s11604-022-01347-1

[17] Lue KH, Chen YH, Chu SC, Lin CB, Wang TF, Liu SH. Prognostic value of combining clinical factors, ^18^F-FDG PET-based intensity, volumetric features, and deep learning predictor in patients with EGFR-mutated lung adenocarcinoma undergoing targeted therapies: a cross-scanner and temporal validation study. Ann Nucl Med. 2024;38:647–58. 38704786 10.1007/s12149-024-01936-2

[18] Wei W, Jia G, Wu Z, Wang T, Wang H, Wei K, Cheng C, Liu Z, Zuo C. A multidomain fusion model of radiomics and deep learning to discriminate between PDAC and AIP based on ^18^F-FDG PET/CT images. Jpn J Radiol. 2023;4:417–27. 10.1007/s11604-022-01363-1 PMC9676903 36409398

[19] Nakajo M, Jinguji M, Ito S, Tani A, Hirahara M, Yoshiura T. Clinical application of ^18^F-fluorodeoxyglucose positron emission tomography/computed tomography radiomics-based machine learning analyses in the field of oncology. Jpn J Radiol. 2024;42:28–55. 37526865 10.1007/s11604-023-01476-1 PMC10764437

[20] Nakajo M, Hirahara D, Jinguji M, Hirahara M, Tani A, Nagano H, et al. Applying deep learning-based ensemble model to [^18^F]-FDG-PET-radiomic features for differentiating benign from malignant parotid gland diseases. Jpn J Radiol. 2025;43:91–100. 39254903 10.1007/s11604-024-01649-6 PMC11717794

[21] Sung C, Oh JS, Park BS, Kim SS, Song SY, Lee JJ. Diagnostic performance of a deep-learning model using ^18^F-FDG PET/CT for evaluating recurrence after radiation therapy in patients with lung cancer. Ann Nucl Med. 2024;38:516–24. 38589677 10.1007/s12149-024-01925-5

[22] Nishimori M, Iwasa H, Nakaji K, Nitta N, Miyatake K, Yoshimatsu R, et al. Predicting the pathological invasiveness of early lung adenocarcinoma prior to surgery using Deauville criteria: reliability and validity. Jpn J Radiol. 2023;41:768–76. 36752955 10.1007/s11604-023-01397-z PMC10313578

[23] Iwasa H, Nagamachi S, Nakayama S, Yamamoto T, Yoshimitsu K. The reproducibility of MTV and TLG of soft tissue tumors calculated by FDG-PET: comparison between the lower limit by the fixed value SUV 2.5 and that value by 30% of SUVmax. Jpn J Radiol. 2023;41:531–40. 36637680 10.1007/s11604-022-01378-8 PMC10147792

[24] Ueki Y, Otsuka H, Otani T, Kasai R, Otomi Y, Ikemitsu D, et al. Combined visual and quantitative assessment of somatostatin receptor scintigraphy for staging and restaging of neuroendocrine tumors. Jpn J Radiol. 2024;42:519–35. 38345724 10.1007/s11604-024-01529-z

[25] Xie Y, Teng Y, Jiang C, Ding C, Zhou Z. Prognostic value of 18F-FDG lesion dissemination features in patients with peripheral T-cell lymphoma (PTCL). Jpn J Radiol. 2023;41:777–86. 36752954 10.1007/s11604-023-01398-y

[26] Yap WK, Hsu KH, Wang TH, Lin CH, Kang CJ, Huang SM, et al. The prognostic value of lymph node to primary tumor standardized uptake value ratio in cancer patients: a meta-analysis. Ann Nucl Med. 2024;38:607–18. 38724805 10.1007/s12149-024-01933-5

[27] Kuroda H, Yoshizako T, Yada N, Kamimura T, Yamamoto N, Maruyama M, et al. Exploration of tumor size measurement methods in preoperative breast cancer assessment using whole-body silicon photomultiplier PET: feasibility and first results. Jpn J Radiol. 2024;42:639–47. 38345725 10.1007/s11604-024-01533-3 PMC11139740

[28] Wumener X, Zhang Y, Zang Z, Ye X, Zhao J, Zhao J, et al. The value of net influx constant based on FDG PET/CT dynamic imaging in the differential diagnosis of metastatic from non-metastatic lymph nodes in lung cancer. Ann Nucl Med. 2024;38:904–12. 39078558 10.1007/s12149-024-01964-y PMC11489159

[29] Yin H, Liu G, Mao W, Lv J, Yu H, Cheng D, et al. Parametric net influx rate imaging of ^68^Ga-DOTATATE in patients with neuroendocrine tumors: assessment of lesion detectability. Ann Nucl Med. 2024;38:483–92. 38573411 10.1007/s12149-024-01922-8

[30] Guner LA, Unal K, Beylergil V, Tuna MB, Saglican Y, Vardareli E, et al. Enhancing PSMA PET/CT imaging of prostate cancer: investigating the impact of multiple time point evaluation, diuretic administration, cribriform pattern, and intraductal carcinoma. Ann Nucl Med. 2023;37:618–28. 37783903 10.1007/s12149-023-01864-7

[31] Burasothikul P, Navikhacheevin C, Pasawang P, Sontrapornpol T, Sukprakun C, Khamwan K. Dual-time-point dynamic ^68^Ga-PSMA-11 PET/CT for parametric imaging generation in prostate cancer. Ann Nucl Med. 2024;38:700–10. 38761312 10.1007/s12149-024-01939-z

[32] Ng QK, Triumbari EKA, Omidvari N, Cherry SR, Badawi RD, Nardo L. Total-body PET/CT - first clinical experiences and future perspectives. Semin Nucl Med. 2022;52:330–9. 35272853 10.1053/j.semnuclmed.2022.01.002 PMC9439875

[33] Zheng Z, He Y, Mao W, Yu H, Wu H, Yang R, et al. Exploration the feasibility and additional value of [^18^F]FDG/[^68^Ga]Ga-FAPI-04 dual-low-activity-tracer one-stop total-body PET imaging at 34 min post-injection of [^68^Ga]Ga-FAPI-04. Eur J Nucl Med Mol Imaging. 2025;52:638–47. 39320482 10.1007/s00259-024-06924-2

[34] Whitfield GA, Kennedy SR, Djoukhadar IK, Jackson A. Imaging and target volume delineation in glioma. Clin Oncol (R Coll Radiol). 2014;26:364–76. 24824451 10.1016/j.clon.2014.04.026

[35] Husby T, Johannessen K, Berntsen EM, Johansen H, Giskeødegård GF, Karlberg A, et al. ^18^F-FACBC and ^18^F-FDG PET/MRI in the evaluation of 3 patients with primary central nervous system lymphoma: a pilot study. EJNMMI Rep. 2024;8:2. 38748286 10.1186/s41824-024-00189-6 PMC10962628

[36] Brendle C, Maier C, Bender B, Schittenhelm J, Paulsen F, Renovanz M, et al. Impact of ^18^F-FET PET/MRI on clinical management of brain tumor patients. J Nucl Med. 2022;63:522–7. 34353870 10.2967/jnumed.121.262051 PMC8973289

[37] Kaneko K, Nagao M, Ueda K, Yamamoto A, Sakai S. Simultaneous evaluation of brain metastasis and thoracic cancer using semiconductor ^11^C-methionine PET/CT imaging. Ann Nucl Med. 2024;38:278–87. 38386272 10.1007/s12149-024-01908-6

[38] Wang Y, Fushimi Y, Arakawa Y, Shimizu Y, Sano K, Sakata A, et al. Evaluation of isocitrate dehydrogenase mutation in 2021 world health organization classification grade 3 and 4 glioma adult-type diffuse gliomas with 18F-fluoromisonidazole PET. Jpn J Radiol. 2023;41:1255–64. 37219717 10.1007/s11604-023-01450-x PMC10613590

[39] Perlow HK, Nalin AP, Handley D, Gokun Y, Blakaj DM, Beyer SJ, et al. A prospective registry study of ^68^ga-dotatate PET/CT incorporation into treatment planning of intracranial meningiomas. Int J Radiat Oncol Biol Phys. 2024;118:979–85. 37871886 10.1016/j.ijrobp.2023.10.014

[40] Watabe T, Naka S, Tatsumi M, Kamiya T, Kimura T, Shintani Y, et al. Initial evaluation of [^18^F]FAPI-74 PET for various histopathologically confirmed cancers and benign lesions. J Nucl Med. 2023;64:1225–31. 37268427 10.2967/jnumed.123.265486 PMC10394310

[41] Arak H, Elboga U, Cayirli YB, Aytekin A. Prognostic significance of 68 Ga-FAPI PET/CT in patients with bone metastases in various cancers. Ann Nucl Med. 2024;38(8):630–8. 10.1007/s12149-024-01935-3. 38684594 10.1007/s12149-024-01935-3

[42] Rao W, Fang XH, Zhao Y, Wang Y, Zhang B, Wei Z, et al. Clinical value of [^18^F]AlF-NOTA-FAPI-04 PET/CT for assessing early-stage liver fibrosis in adult liver transplantation recipients compared with chronic HBV patients. Jpn J Radiol. 2024;42:536–45. 38316724 10.1007/s11604-024-01528-0

[43] Mori Y, Tamburini K, Novruzov E, Schmitt D, Mavriopoulou E, Loosen SH, et al. Efficacy of [^68^Ga]Ga-FAPI-PET as a non-invasive evaluation method of liver fibrosis. Ann Nucl Med. 2025;39:631–9. 40048016 10.1007/s12149-025-02027-6 PMC12095406

[44] Hofman MS, Lawrentschuk N, Francis RJ, Tang C, Vela I, Thomas P, et al. Prostate-specific membrane antigen PET-CT in patients with high-risk prostate cancer before curative-intent surgery or radiotherapy (proPSMA): a prospective, randomised, multicentre study. Lancet. 2020;395:1208–16. 32209449 10.1016/S0140-6736(20)30314-7

[45] Otani T, Nakamoto R, Umeoka S, Nakamoto Y. PSMA PET/CT imaging and its application to prostate cancer treatment. Jpn J Radiol. 2025;43:1–12. 39225954 10.1007/s11604-024-01646-9 PMC11717842

[46] Watabe T, Uemura M, Soeda F, Naka S, Ujike T, Hatano K, et al. High detection rate in [^18^F]PSMA-1007 PET: interim results focusing on biochemical recurrence in prostate cancer patients. Ann Nucl Med. 2021;35:523–8. 33661475 10.1007/s12149-021-01602-x PMC7981319

[47] Tateishi U, Kimura K, Tsuchiya J, Kano D, Watabe T, Nonomura N, et al. Phase I/IIa trial of 18F-prostate specific membrane antigen (PSMA) 1007 PET/CT in healthy volunteers and prostate cancer patients. Jpn J Clin Oncol. 2024;54:282–91. 38066703 10.1093/jjco/hyad166

[48] Cardinale J, Giesel FL, Wensky C, Rathke HG, Haberkorn U, Kratochwil C. PSMA-GCK01: a generator-based ^99m^Tc/^188^Re theranostic ligand for the prostate-specific membrane antigen. J Nucl Med. 2023;64:1069–75. 36759199 10.2967/jnumed.122.264944 PMC10315696

[49] Shimizu Y, Ando M, Watanabe H, Ono M. Novel technetium-99m-labeled bivalent PSMA-targeting probe based on hydroxamamide chelate for diagnosis of prostate cancer. Ann Nucl Med. 2024;38:847–51. 38976087 10.1007/s12149-024-01959-9

[50] Pouliot F, Saad F, Rousseau E, Richard PO, Zamanian A, Probst S, et al. intrapatient intermetastatic heterogeneity determined by triple-tracer PET imaging in mCRPC patients and correlation to survival: the 3TMPO cohort study. J Nucl Med. 2024;65:1710–7. 39327017 10.2967/jnumed.124.268020 PMC11533914

[51] Belge Bilgin G, Bilgin C, Orscelik A, Burkett BJ, Thorpe MP, Johnson DR, et al. Detection rate of gastrin-releasing peptide receptor (GRPr) targeted tracers for positron emission tomography (PET) imaging in primary prostate cancer: a systematic review and meta-analysis. Ann Nucl Med. 2024;38:865–76. 39287742 10.1007/s12149-024-01978-6

[52] Ulaner GA, Jhaveri K, Chandarlapaty S, Hatzoglou V, Riedl CC, Lewis JS, Mauguen A. Head-to-head evaluation of ^18^F-FES and ^18^F-FDG PET/CT in metastatic invasive lobular breast cancer. J Nucl Med. 2021;62:326–31. 32680923 10.2967/jnumed.120.247882 PMC8049349

[53] Liu C, Ma G, Zhang J, Cheng J, Yang Z, Song S. ^18^F-FES and ^18^F-FDG PET/CT imaging as a predictive biomarkers for metastatic breast cancer patients undergoing cyclin-dependent 4/6 kinase inhibitors with endocrine treatment. Ann Nucl Med. 2023;37:675–84. 37787851 10.1007/s12149-023-01871-8

[54] Chan DL, Hayes AR, Karfis I, Conner A, Furtado O’Mahony L, Mileva M, et al. Dual [^68^Ga]DOTATATE and [^18^F]FDG PET/CT in patients with metastatic gastroenteropancreatic neuroendocrine neoplasms: a multicentre validation of the NETPET score. Br J Cancer. 2023;128:549–55. 36434154 10.1038/s41416-022-02061-5 PMC9938218

[55] Strosberg JR, Caplin ME, Kunz PL, Ruszniewski PB, Bodei L, Hendifar A, et al. ^177^Lu-Dotatate plus long-acting octreotide versus high-dose long-acting octreotide in patients with midgut neuroendocrine tumours (NETTER-1): final overall survival and long-term safety results from an open-label, randomised, controlled, phase 3 trial. Lancet Oncol. 2021;22:1752–63. 34793718 10.1016/S1470-2045(21)00572-6

[56] Singh S, Halperin D, Myrehaug S, Herrmann K, Pavel M, Kunz PL, et al. [^177^Lu]Lu-DOTA-TATE plus long-acting octreotide versus high-dose long-acting octreotide for the treatment of newly diagnosed, advanced grade 2–3, well-differentiated, gastroenteropancreatic neuroendocrine tumours (NETTER-2): an open-label, randomised, phase 3 study. Lancet. 2024;403:2807–17. 38851203 10.1016/S0140-6736(24)00701-3

[57] Hotta M, Sonni I, Thin P, Nguyen K, Gardner L, Ciuca L, et al. Visual and whole-body quantitative analyses of ^68^Ga-DOTATATE PET/CT for prognosis of outcome after PRRT with ^177^Lu-DOTATATE. Ann Nucl Med. 2024;38:296–304. 38252228 10.1007/s12149-023-01899-w

[58] Calderoni L, Maietti E, Farolfi A, Mei R, Louie KS, Groaning M, et al. Prostate-specific membrane antigen expression on PET/CT in patients with metastatic castration-resistant prostate cancer: a retrospective observational study. J Nucl Med. 2023;64:910–7. 36635087 10.2967/jnumed.122.264964 PMC10241018

[59] Hofman MS, Emmett L, Sandhu S, Iravani A, Joshua AM, Goh JC, et al. [^177^Lu]Lu-PSMA-617 versus cabazitaxel in patients with metastatic castration-resistant prostate cancer (TheraP): a randomised, open-label, phase 2 trial. Lancet. 2021;397:797–804. 33581798 10.1016/S0140-6736(21)00237-3

[60] Sartor O, de Bono J, Chi KN, Fizazi K, Herrmann K, Rahbar K, et al. Lutetium-177-PSMA-617 for metastatic castration-resistant prostate cancer. N Engl J Med. 2021;385:1091–103. 34161051 10.1056/NEJMoa2107322 PMC8446332

[61] Morris MJ, Castellano D, Herrmann K, de Bono JS, Shore ND, Chi KN, et al. ^177^Lu-PSMA-617 versus a change of androgen receptor pathway inhibitor therapy for taxane-naive patients with progressive metastatic castration-resistant prostate cancer (PSMAfore): a phase 3, randomised, controlled trial. Lancet. 2024;404:1227–39. 39293462 10.1016/S0140-6736(24)01653-2 PMC12121614

[62] Michalski K, Kosmala A, Werner RA, Serfling SE, Seitz AK, Lapa C, et al. Comparison of PET/CT-based eligibility according to VISION and TheraP trial criteria in end-stage prostate cancer patients undergoing radioligand therapy. Ann Nucl Med. 2024;38:87–95. 37891376 10.1007/s12149-023-01874-5 PMC10822822

[63] Higuchi T, Klimek K, Groener D, Chen X, Werner RA. Norepinephrine transporter-targeted cancer theranostics-new horizons. Clin Nucl Med. 2025;50:44–51. 39570057 10.1097/RLU.0000000000005567

[64] Kuroda R, Wakabayashi H, Araki R, Inaki A, Nishimura R, Ikawa Y, et al. Phase I/II clinical trial of high-dose [^131^I] meta-iodobenzylguanidine therapy for high-risk neuroblastoma preceding single myeloablative chemotherapy and haematopoietic stem cell transplantation. Eur J Nucl Med Mol Imaging. 2022;49:1574–83. 34837510 10.1007/s00259-021-05630-7

[65] Haugen BR, Alexander EK, Bible KC, Doherty GM, Mandel SJ, Nikiforov YE, et al. 2015 American thyroid association management guidelines for adult patients with thyroid nodules and differentiated thyroid cancer: the american thyroid association guidelines task force on thyroid nodules and differentiated thyroid cancer. Thyroid. 2016;26:1–133. 26462967 10.1089/thy.2015.0020 PMC4739132

[66] Weis H, Weindler J, Schmidt K, Hellmich M, Drzezga A, Schmidt M. Impact of radioactive iodine treatment on long-term relative survival in patients with papillary and follicular thyroid cancer: a SEER-based study covering histologic subtypes and recurrence risk categories. J Nucl Med. 2025;66:525–30. 40081954 10.2967/jnumed.124.269091 PMC11960602

[67] Zhang Y, Zhu X, Fan Q, Huang Q, Tu Y, Jiang L, Zhang Z, Chen J. Utility of adjuvant radioactive iodine therapy after reoperation in papillary thyroid carcinoma with cervical lymph node recurrence. Jpn J Radiol. 2023;41:1148–56. 37266825 10.1007/s11604-023-01438-7

[68] Watanabe K, Igarashi T, Uchiyama M, Ishigaki T, Ojiri H. Retrospective case-control study examining the relationship between recurrence-free survival and changes in pre- and post-radioiodine therapy serum thyroglobulin levels in patients with differentiated thyroid cancer. Jpn J Radiol. 2024;42:391–7. 38212512 10.1007/s11604-023-01517-9

[69] Dai X, Ren X, Zhang J, Zheng Y, Wang Z, Cheng G. Advances in the selection and timing of postoperative radioiodine treatment in patients with differentiated thyroid carcinoma. Ann Nucl Med. 2024;38:688–99. 39044048 10.1007/s12149-024-01963-z

[70] Higashi T, Nishii R, Yamada S, Nakamoto Y, Ishizu K, Kawase S, et al. Delayed initial radioactive iodine therapy resulted in poor survival in patients with metastatic differentiated thyroid carcinoma: a retrospective statistical analysis of 198 cases. J Nucl Med. 2011;52:683–9. 21498534 10.2967/jnumed.110.081059

[71] Resch S, Ziegler SI, Sheikh G, Unterrainer LM, Zacherl MJ, Bartenstein P, et al. Impact of the reference multiple-time-point dosimetry protocol on the validity of single-time-point dosimetry for [^177^Lu]Lu-PSMA-I&T therapy. J Nucl Med. 2024;65:1272–8. 38936975 10.2967/jnumed.123.266871 PMC11294067

[72] Handayani W, Chantadisai M, Phromphao B, Noipinit N, Pasawang P, Khamwan K. Comparative post-therapeutic dosimetry between 2D planar-based and hybrid-based methods for personalized Lu-177 treatment. Ann Nucl Med. 2024;38:884–93. 39023826 10.1007/s12149-024-01960-2

[73] Ono T, Ichikawa M, Tanada T, Kanezawa C, Sato H. Maximum tumor diameter and renal function can predict the declining surface dose rate after ^177^Lu-Dotatate: preliminary results of single institution in Japan. Jpn J Radiol. 2024;42:1031–7. 38727960 10.1007/s11604-024-01585-5 PMC11364653

[74] Naskar N, Lahiri S. Theranostic terbium radioisotopes: challenges in production for clinical application. Front Med (Lausanne). 2021;8: 675014. 34136508 10.3389/fmed.2021.675014 PMC8200528

[75] Buchanan CMJ, Aboagye EO, Evitts LJ, Rushton MJD, Smith TAD. Modelling potential candidates for targeted auger therapy. Biophysica. 2024;4:711–23.

[76] Müller C, Umbricht CA, Gracheva N, Tschan VJ, Pellegrini G, Bernhardt P, et al. Terbium-161 for PSMA-targeted radionuclide therapy of prostate cancer. Eur J Nucl Med Mol Imaging. 2019;46:1919–30. 31134301 10.1007/s00259-019-04345-0 PMC6820371

[77] Borgna F, Haller S, Rodriguez JMM, Ginj M, Grundler PV, Zeevaart JR, et al. Combination of terbium-161 with somatostatin receptor antagonists-a potential paradigm shift for the treatment of neuroendocrine neoplasms. Eur J Nucl Med Mol Imaging. 2022;49:1113–26. 34625828 10.1007/s00259-021-05564-0 PMC8921065

[78] Al-Ibraheem A, Doudeen RM, Juaidi D, Abufara A, Maus S. ^161^Tb-PSMA radioligand therapy: first-in-humans SPECT/CT imaging. J Nucl Med. 2023;64:1322–3. 36759201 10.2967/jnumed.122.265291

[79] Fricke J, Westerbergh F, McDougall L, Favaretto C, Christ E, Nicolas GP, et al. First-in-human administration of terbium-161-labelled somatostatin receptor subtype 2 antagonist ([^161^Tb]Tb-DOTA-LM3) in a patient with a metastatic neuroendocrine tumour of the ileum. Eur J Nucl Med Mol Imaging. 2024;51:2517–9. 38448550 10.1007/s00259-024-06641-w PMC11178597

[80] Kratochwil C, Bruchertseifer F, Giesel FL, Weis M, Verburg FA, Mottaghy F, et al. 225Ac-PSMA-617 for PSMA-targeted α-radiation therapy of metastatic castration-resistant prostate cancer. J Nucl Med. 2016;57:1941–4. 27390158 10.2967/jnumed.116.178673

[81] Parker C, Nilsson S, Heinrich D, Helle SI, O’Sullivan JM, Fosså SD, et al. Alpha emitter radium-223 and survival in metastatic prostate cancer. N Engl J Med. 2013;369:213–23. 23863050 10.1056/NEJMoa1213755

[82] Tombal B, Choudhury A, Saad F, Gallardo E, Soares A, Loriot Y, et al. Enzalutamide plus radium-223 in metastatic castration-resistant prostate cancer: results of the EORTC 1333/PEACE-3 trial. Ann Oncol. 2025;S0923–7534(25):00203. 10.1016/j.annonc.2025.05.011 40450503

[83] Gillessen S, Tombal B, Turco F, Choudhury A, Rodriguez-Vida A, Gallardo E, et al. Decrease in fracture rate with mandatory bone-protecting agents in the EORTC 1333/PEACE-3 trial comparing radium-223 combined with enzalutamide versus enzalutamide alone: a safety analysis. Eur Urol. 2025;87:285–8. 39827019 10.1016/j.eururo.2024.11.027

[84] Tosato M, Favaretto C, Kleynhans J, Burgoyne AR, Gestin JF, van der Meulen NP, et al. Alpha Atlas: Mapping global production of α-emitting radionuclides for targeted alpha therapy. Nucl Med Biol. 2025. 10.1016/j.nucmedbio.2024.108990. 39809026 10.1016/j.nucmedbio.2024.108990

[85] Ballal S, Yadav MP, Tripathi M, Sahoo RK, Bal C. Survival outcomes in metastatic gastroenteropancreatic neuroendocrine tumor patients receiving concomitant ^225^Ac-DOTATATE targeted alpha therapy and capecitabine: a real-world scenario management based long-term outcome study. J Nucl Med. 2023;64:211–8. 10.2967/jnumed.122.264043 35863893

[86] Lee DY, Kim YI. Effects of ^225^Ac-labeled prostate-specific membrane antigen radioligand therapy in metastatic castration-resistant prostate cancer: a meta-analysis. J Nucl Med. 2022;63:840–6. 34503960 10.2967/jnumed.121.262017

[87] Sathekge MM, Lawal IO, Bal C, Bruchertseifer F, Ballal S, Cardaci G, et al. Actinium-225-PSMA radioligand therapy of metastatic castration-resistant prostate cancer (WARMTH Act): a multicentre, retrospective study. Lancet Oncol. 2024;25:175–83. 38218192 10.1016/S1470-2045(23)00638-1

[88] Watabe T, Hosono M, Kinuya S, Yamada T, Yanagida S, Namba M, et al. Manual on the proper use of sodium astatide ([^211^At]NaAt) injections in clinical trials for targeted alpha therapy (1st edition). Ann Nucl Med. 2021;35:753–766. 10.1007/s12149-021-01619-2 PMC8197710 33978932

[89] Watabe T, Hatano K, Naka S, Sasaki H, Kamiya T, Shirakami Y, et al. First-in-human SPECT/CT imaging of [^211^At]PSMA-5: targeted alpha therapy in a patient with refractory prostate cancer. Eur J Nucl Med Mol Imaging. 2025;52:2253–5. 39688698 10.1007/s00259-024-07017-w PMC12119378

[90] Watabe T, Mukai K, Naka S, Sasaki H, Kamiya T, Fukuhara A, et al. J Nucl Med. 2025;66(supplement 1): 251278.

[91] Takacova M, Kajanova I, Kolarcikova M, Lapinova J, Zatovicova M, Pastorekova S. Understanding metabolic alterations and heterogeneity in cancer progression through validated immunodetection of key molecular components: a case of carbonic anhydrase IX. Cancer Metastasis Rev. 2021;40:1035–53. 35080763 10.1007/s10555-021-10011-5 PMC8825433

[92] Nakaigawa N, Hasumi H, Utsunomiya D, Yoshida K, Ishiwata Y, Oka T, et al. Evaluation of PET/CT imaging with [89Zr]Zr-DFO-girentuximab: a phase 1 clinical study in Japanese patients with renal cell carcinoma (Zirdac-JP). Jpn J Clin Oncol. 2024;54:873–9. 38864246 10.1093/jjco/hyae075 PMC11322881

[93] Hofman MS, Tran B, Feldman DR, Pokorska-Bocci A, Pichereau S, Wessen J, et al. First-in-human safety, imaging, and dosimetry of a carbonic anhydrase ix-targeting peptide, [^68^Ga]Ga-DPI-4452, in patients with clear cell renal cell carcinoma. J Nucl Med. 2024;65:740–3. 38388517 10.2967/jnumed.123.267175 PMC11064824

[94] Lenárt S, Lenárt P, Šmarda J, Remšík J, Souček K, Beneš P. Trop2: jack of all trades, master of none. Cancers (Basel). 2020;12:3328. 33187148 10.3390/cancers12113328 PMC7696911

[95] Liao Q, Zhang R, Ou Z, Ye Y, Zeng Q, Wang Y, et al. TROP2 is highly expressed in triple-negative breast cancer CTCs and is a potential marker for epithelial mesenchymal CTCs. Mol Ther Oncol. 2024;32: 200762. 38596285 10.1016/j.omton.2024.200762 PMC10869581

[96] Chen H, Zhao L, Pang Y, Shi J, Gao H, Sun Y, et al. 68Ga-MY6349 PET/CT imaging to assess Trop2 expression in multiple types of cancer. J Clin Invest. 2024;135: e185408. 39509246 10.1172/JCI185408 PMC11684813

[97] Wu Y, Li T, Zhang X, Jing H, Li F, Huo L. Preclinical evaluation of the theranostic potential of ^89^Zr/^177^Lu-labeled anti-TROP-2 antibody in triple-negative breast cancer model. EJNMMI Radiopharm Chem. 2024;9:5. 38194043 10.1186/s41181-023-00235-x PMC10776551

[98] Hashimoto H, Tanaka Y, Murata M, Ito T. Nectin-4: a novel therapeutic target for skin cancers. Curr Treat Options Oncol. 2022;23:578–93. 35312963 10.1007/s11864-022-00940-w

[99] Powles T, Rosenberg JE, Sonpavde GP, Loriot Y, Durán I, Lee JL, Matsubara N, et al. Enfortumab vedotin in previously treated advanced urothelial carcinoma. N Engl J Med. 2021;384:1125–35. 33577729 10.1056/NEJMoa2035807 PMC8450892

[100] Duan X, Xia L, Zhang Z, Ren Y, Pomper MG, Rowe SP, et al. First-in-human study of the radioligand 68GA-N188 targeting Nectin-4 for PET/CT imaging of advanced urothelial carcinoma. Clin Cancer Res. 2023;29:3395–407. 37093191 10.1158/1078-0432.CCR-23-0609

[101] Zhang J, Duan X, Chen X, Zhang Z, Sun H, Shou J, et al. Translational PET imaging of Nectin-4 expression in multiple different cancers with ^68^Ga-N188. J Nucl Med. 2024;65(Suppl 1):12S-18S. 38719240 10.2967/jnumed.123.266830

[102] Kaneda-Nakashima K, Shirakami Y, Hisada K, Feng S, Kadonaga Y, Ooe K, et al. Development of LAT1-selective nuclear medicine therapeutics using astatine-211. Int J Mol Sci. 2024;25:12386. 39596451 10.3390/ijms252212386 PMC11594329

[103] Watabe T, Kabayama K, Naka S, Yamamoto R, Kaneda K, Serada S, et al. Immuno-PET and targeted α-therapy using anti-glypican-1 antibody labeled with ^89^Zr or ^211^at: a theranostic approach for pancreatic ductal adenocarcinoma. J Nucl Med. 2023;64:1949–55. 37827841 10.2967/jnumed.123.266313 PMC10690121

[104] Watabe T, Iwasawa T, Kimura H, Shirakami Y, Naka S, Kaneda K, et al. Theranostics using ^89^Zr/^177^Lu-labeled antibody targeting erythropoietin-producing hepatocellular A2 (EphA2). Eur J Nucl Med Mol Imaging. 2025;52:2887–97. 39934299 10.1007/s00259-025-07139-9 PMC12162727

